# REM-OSA as a Tool to Understand Both the Architecture of Sleep and Pathogenesis of Sleep Apnea—Literature Review

**DOI:** 10.3390/jcm12185907

**Published:** 2023-09-12

**Authors:** Filip Franciszek Karuga, Piotr Kaczmarski, Piotr Białasiewicz, Bartosz Szmyd, Julia Jaromirska, Filip Grzybowski, Piotr Gebuza, Marcin Sochal, Agata Gabryelska

**Affiliations:** 1 Department of Sleep Medicine and Metabolic Disorders, Medical University of Lodz, Mazowiecka St. 6/8, 92-251 Lodz, Polandfilip.grzybowski@stud.umed.lodz.pl (F.G.);; 2 Department of Pediatrics, Oncology and Hematology, Medical University of Lodz, Sporna St. 36/50, 91-738 Lodz, Poland; 3 Department of Neurosurgery and Neuro-Oncology, Medical University of Lodz, Barlicki University Hospital, Kopcinskiego St. 22, 90-153 Lodz, Poland

**Keywords:** OSA, phenotype, REM, sleep architecture, CPAP

## Abstract

Sleep is a complex physiological state, which can be divided into the non-rapid eye movement (NREM) phase and the REM phase. Both have some unique features and functions. This difference is best visible in electroencephalography recordings, respiratory system activity, arousals, autonomic nervous system activity, or metabolism. Obstructive sleep apnea (OSA) is a common condition characterized by recurrent episodes of pauses in breathing during sleep caused by blockage of the upper airways. This common condition has multifactorial ethiopathogenesis (e.g., anatomical predisposition, sex, obesity, and age). Within this heterogenous syndrome, some distinctive phenotypes sharing similar clinical features can be recognized, one of them being REM sleep predominant OSA (REM-OSA). The aim of this review was to describe the pathomechanism of REM-OSA phenotype, its specific clinical presentation, and its consequences. Available data suggest that in this group of patients, the severity of specific cardiovascular and metabolic complications is increased. Due to the impact of apneas and hypopneas predominance during REM sleep, patients are more prone to develop hypertension or glucose metabolism impairment. Additionally, due to the specific function of REM sleep, which is predominantly fragmented in the REM-OSA, this group presents with decreased neurocognitive performance, reflected in memory deterioration, and mood changes including depression. REM-OSA clinical diagnosis and treatment can alleviate these outcomes, surpassing the traditional treatment and focusing on a more personalized approach, such as using longer therapy of continuous positive airway pressure or oral appliance use.

## 1. Introduction

Sleep is a complex physiological state of reduced responsiveness to external stimuli and easily reversed loss of consciousness. Modern sleep-tracking devices brought into general use, such as wearable sensors or mobile sleep-monitoring apps, provide data demonstrating 7.27 h as a mean value of sleep duration for women and 7 h for men [1]. However, as the duration varies from person to person on account of numerous variables, including age, body mass index, medical status, or heritability, defining the universal optimal time spent on sleeping remains impossible [2,3,4,5]. Average sleep consists of four to six sleep cycles, which reflect the impact of ultradian rhythm; within one cycle, non-rapid eye movement (NREM) and rapid eye movement (REM) sleep can be distinguished. Despite being ubiquitous throughout one’s whole life, sleep is still widely regarded as a mysterious process, whose function in maintaining homeostasis is not apparent as it might appear at first sight and is not precisely identified. Sleep is regulated by the interplay of the circadian cycle and sleep homeostasis, independently determining sleep and sleep-related processes [6]. Circadian cycles are repetitive 24 h of sleep–wake patterns, that are kept under the control of the suprachiasmatic nuclei of the hypothalamus (commonly called the master circadian pacemaker), and provide functional adjustment to the light-dark cycle. Homeostatic sleep changes are provided by enhanced sleep pressure that strengthens during waking hours (and presumably during REM sleep). Poor sleep quality may lead to immediately noticeable detrimental consequences, such as heightened stress response or somatic symptoms including frequent headaches and abdominal discomfort. Mental issues, commonly in the form of severe emotional stress and cognitive deficits, are also considered as the outcome of sleep disturbances. Additionally, prolonged sleep deprivation increases the likelihood of obesity, cardiovascular disease, diabetes mellitus, or cancer development and, therefore, a risk of all-cause mortality [7].

Sleep apnea is the second most common sleep disorder after insomnia among adults. It is characterized by repetitive apneas and hypopneas during sleep, which can result either from respiratory center signaling disruption—central sleep apnea (CSA) or from temporary complete/partial upper airway collapse—obstructive sleep apnea (OSA) [8]. The apnea–hypopnea index (AHI) is the total average number of apneas and hypopneas per hour of sleep, which is used as a scale to identify the severity of OSA with the relevance: 15 > AHI ≥ 5—mild, 30 > AHI ≥ 15—moderate, AHI ≥ 30—severe [9]. Moderate to severe OSA with an estimated global prevalence of approximately 20% in the general population is substantially more common than CSA [10]. The gold standard for OSA diagnosis is overnight polysomnography (PSG), which is a multiparameter examination of objective and measurable sleep quality. Risk factors for OSA encompass male gender, middle age, overweight/obesity, craniofacial and upper airway abnormalities, circadian clock disruption, and familial aggregation [11,12]. Due to the non-specific and indefinite value of the most common manifestations (including snoring, excessive daytime sleepiness, and morning headaches), the disease is difficult to detect. The most efficacious and evaluated OSA treatment is continuous positive airway pressure (CPAP), which is based on maintaining constant upper airway patency [13]. Apart from CPAP, there are a number of alternative treatment options for OSA patients, including oral appliances designed to keep the patency of upper airways, positional therapy that prevents patients from sleeping in a supine position, stimulation of hypoglossal nerve that enables the control of tongue movement, and other surgical techniques leading to improvement of upper airway anatomy [14]. Untreated OSA may contribute to serious complications—resulting from both nocturnal hypoxia and decreased sleep architecture—including disrupted glucose metabolism, diabetes mellitus, metabolic syndrome, cardiovascular diseases, and neurocognitive impairment [15,16,17,18,19,20]. The heterogeneity of the OSA clinical picture creates the opportunity to distinguish phenotypes, thereby improving both diagnostics and treatment options, focusing on a more personalized approach [21]. The most commonly used and best-described types include REM-predominant OSA or stage-independent OSA, in accordance with the aforementioned AHI value in particular phases.

We conducted a review of current literature regarding sleep stages, OSA, and phenotyping of OSA through a search of the following databases: PubMed (search queries: “NREM sleep”, “REM sleep”, “OSA and phenotype”, “REM and OSA”, “REM predominant OSA”, “REM and OSA and CPAP”) and Google Scholar (search queries: “REM OSA”, “NREM OSA”, “REM OSA polysomnography”, “NREM sleep”, “REM sleep”, “REM OSA and hypertension”, “REM OSA and diabetes mellitus”, “OSA and phenotype”, “REM OSA and treatment”, “hypnogram”). Additionally, relevant publications from references of examined reports were included.

This review concerns the influence of abnormal sleep architecture on OSA pathogenesis in the example of REM-predominant OSA phenotype and is aimed at demonstrating dependencies between the sleep stage of apnea appearance and clinical features of the disease.

## 2. Sleep Architecture

**Figure 1:**
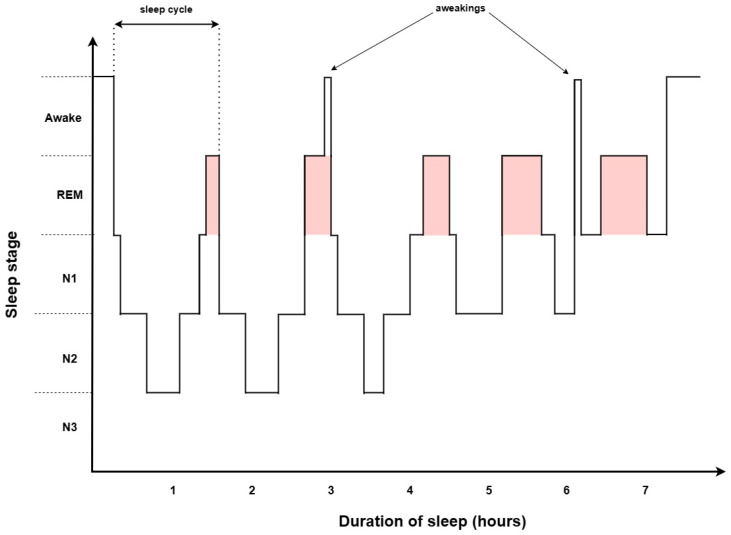
Standard hypnogram. The visualization of the cyclical pattern of sleep stages. The sleep cycle is composed of two phases: rapid eye movement sleep (REM), which accounts for 25% of sleep, and non-rapid eye movement sleep (NREM), which encompasses three stages (N1, N2, N3) and constitutes approximately 75% of sleep. The average sleep cycle lasts 90–120 min, physiological sleep consists of four to six sleep cycles per night. The first cycle is the shortest and lasts 70–100 min, REM phase duration (marked in red color) elongates, while NREM sleep shortens in each cycle during sleep.

Sleep is composed of two distinct brain activity states, NREM and REM, which appear in a cyclical pattern and play complementary roles in recovery after the waking period (see Figure 1) [22]. They differ in brain activity, eye movements, and muscle activity, reflected in PSG parameters—electroencephalography (EEG), electrooculography (EOG), and electromyography (EMG)—which define temporal sleep dynamics. An average first sleep cycle lasts 70–100 min in contrast to the subsequent 90–120 min cycles. Overall, NREM sleep constitutes approximately 75% of nightly sleep; however, the NREM phase shortens sleep time, whereas REM sleep elongates [23]. Significant differences between NREM and REM sleep in both structure and function indicate a diverse response to their deprivation, and show the promising clinical importance of phase-associated OSA phenotypes.

### 2.1. NREM

NREM sleep starts at the onset of sleep and encompasses three stages (N1, N2, N3) according to the current American Academy of Sleep Medicine nomenclature, wherein stage N3 reflects the former stages three and four established by Rechtschaffen and Kales [24,25].

The first stage of sleep—N1 is preceded by the wake stage, or stage W, in which the occipital alpha rhythm (8–13 Hz) prevails; however, in the states of increased alertness, beta waves (>13 Hz) are significant. In the case of the absence of alpha waves (which occurs in up to 20% of patients), stage W determination is supported by the presence of eye blinking with 0.5–2 Hz frequency, or rapid eye movement with a simultaneous normal/increased chin EMG tone. Stage N1, as a transitional stage, lasts roughly 5% of sleep and tends to be more expanded in men, causing more nocturnal awakenings and, therefore, explaining the increased tendency to daytime sleepiness complaints [26]. In general, the stage is characterized by low-amplitude, mixed-frequency pattern, slow eye movements (predominantly lasting >500 ms), and lower muscle tonus compared to wakefulness. Additionally, in EEG, record vertex sharp waves can be noticeable. Stage N1 is classified as light sleep, the prevalent waves are theta (4–8 Hz) and attenuated alpha, which provide the bridge between consciousness and deeper sleep.

K-complex (a non-specific evoked response with a frequency <1 Hz) and sleep spindle (a burst of 10–15 Hz activity) are hallmarks of the second stage of NREM (N2) onset. However, the presence of K-complex with the following arousal does not indicate the start of the N2 epoch. About 45% of sleep falls within the N2 stage and its duration, as the only NREM stage, gradually expands throughout the night. Overall, N1 and N2 stages are classified as light sleep and their duration along with stage W increase with age starting from adolescence.

In the third stage—N3, at least 20% of the brain activity is described as slow wave activity (0.5–2 Hz frequency and >75 µV peak-to-nadir voltage). The main brain waves are theta and delta (0–4 Hz), in which power frequently slows with age [27]. Aging contributes to the reduction in the overall time spent in stage N3, as well [28]. Records of eye movement and muscle tonus exhibit the lowest values among NREM stages. Stage N3 makes up 25% of sleep and contributes to the deepest NREM sleep followed by the REM phase.

EEG record of OSA during NREM in comparison to healthy subjects is characterized by substantial enhancement of alpha and beta wave frequency and diminution of low (0–2 Hz) and high (2–4 Hz) delta band [29,30]. Moreover, NREM beta EEG power correlates with the severity of the disease—the greater AHI is, the higher beta power can be detected [31]. The duration of the N1 stage is longer, whereas N3 is shorter. Stage N3 sometimes ameliorates disturbed breathing by maintaining greater upper airway stability, likely due to the increased phasic electromyographic activity of pharyngeal muscles [32,33]. The alternative explanation relies on the higher arousal threshold in stage N3, which reduces the number of arousals [34]. However, usually stage N3 is highly unstable during exposure to obstructive episodes and transition to light sleep ensues. These dependencies may be also associated with age and gender—during stage N3, females experience less obstructive episodes than males, and males ≥40 years have more respiratory events than other males; in females, AHI does not significantly differ across age groups [35]. Response to CPAP treatment results in K-complex frequency reduction during stage N2, and during stage N3, activity increases [36].

The influence of particularly NREM sleep on the respiratory system remains noticeable. NREM sleep decreases the tidal volume and decreases or does not affect the respiratory rate (RR). As a result, minute ventilation declines, and partial pressure of carbon dioxide slightly increases [37,38,39]. In comparison to wakefulness and REM sleep, respiration during NREM sleep stabilizes [40]. The respiratory effort amplitude is more regular in NREM than in wakefulness and REM sleep, which makes respiration more rhythmical and stable despite slight fluctuations [41,42]. Breathing during NREM sleep is mostly controlled by metabolic feedback, not extrinsic or behavioral stimuli [43]. Upper airway muscles, such as genioglossus or tensor palatini, exhibit increased activity with the transition from light NREM to deep NREM sleep—their tonus may be partly regulated by stimulation of mechanoreceptors located in upper airways and central chemoreceptors [44]. However, isolated hypercapnia cannot activate upper airway muscle during stage N3, which stands in opposition to animal models [45,46,47]. Upper airway mechanoreceptors also have a diminished effect on muscles during NREM compared to wakefulness and REM sleep [48]. In turn, arousal from NREM sleep causes ventilation increase in the first two breaths due to the increase in tidal volume and respiratory rate [49]. Gender determines breathing variability in NREM sleep through differences in chemoresponsiveness—women tend to have a lower hypocapnic apneic threshold and more stable breathing. In addition, the response to inspiratory resistive load is lower and pharyngeal resistance is enhanced in males compared to females [50,51]. The marked differences can result from varying levels of testosterone, which increase the hypocapnic apneic threshold, decrease the arousal threshold, and lead to greater susceptibility to apnea in men [52,53,54]. Interestingly, progesterone and estrogen have demonstrated a disparate impact on NREM sleep breathing [55,56]. Respiratory chemosensitivity during NREM is decreased and may depend on age—the highest outcomes are obtained from the elderly, as demonstrated by increased isocapnic hypoxic ventilatory response and hyperoxic suppression. It might be the reason for the increased incidence of CSA and periodic breathing in the NREM phase amongst older people [57,58]. NREM-dependent OSA has been described in the literature as OSA phenotype with higher propensity of respiratory events during NREM sleep. There is still little data on how to diagnose and recognize NREM-dependent OSA, as the diagnostic criteria used in studies regarding this phenotype are not consistent. It was suggested that NREM-dependent OSA may be associated with greater total AHI, arousal index, and breathing instability than REM-predominant OSA; however, the potential clinical significance of NREM-predominant OSA has not been discovered yet [59,60,61,62,63,64].

The cardiovascular system undergoes profound modifications during NREM sleep, as well. Heart rate variability during NREM is associated with high-frequency (>0.15 Hz) activity increase and low-frequency (0.04–0.15 Hz) activity decrease, which results from decrease in sympathetic activity after the end of the REM phase, and has been related to the general dominance of the parasympathetic nervous system [65]. Enhanced vagal activity reduces blood pressure and heart rate, which positively impacts cardiovascular health. The most noteworthy drop occurs during stage N3 [66]. However, in stage N1, transitory sympathetic increase and heart rate rise are observed [67]. During NREM sleep, brain and heart oscillations interrelate with each other, the stronger and more precise slow oscillations and sleep spindles are, the better parasympathetic control over the cardiovascular system is [68]. Aging may undermine vagal dominance in NREM sleep, and thus impair cardiovascular stability, which leads to poorer sleep quality and the development of cardiovascular disease [69,70]. The cardiovascular system’s response to NREM sleep might also depend on race and ethnicity [71,72]. Arousal from NREM sleep is associated with vasoconstriction, mean arterial pressure and heart rate increase, and cerebral blood flow velocity decrease with progressive return to the state preceding the arousal [49,73]. Cerebrovascular reactivity in response to hypercapnia during NREM sleep declines, which may lead to reduced cerebral blood flow [74]. Interestingly, daytime naps have an equivalent beneficial impact on the cardiovascular system during NREM compared to nocturnal NREM sleep [75,76]. Sleep apnea-related NREM cardiovascular changes include noteworthy blood pressure and heart rate fluctuations; the largest rise is noticeable between the first and last third of the obstructive event [77,78,79,80]. Similarly, QT interval variability exhibits higher mean values in comparison to non-OSA patients [81]. 

As noted, respiratory and cardiovascular changes occur largely under control of the autonomic nervous system. Increased vagal activity reduces vascular tone and muscle tonus, providing favorable conditions for energy recovery [82]. Autonomic activity in NREM sleep influences almost every body function, including thermoregulation. Enhanced heat loss in response to vasodilation induces a decrease in body temperature during NREM and results in lower metabolic activity [83].

Under current knowledge, the main function of NREM sleep aims at restorative processes in the whole body, but especially in the brain. Synaptic renormalization and cellular maintenance may exist due to the remodeling of cortical plasticity within slow wave sleep [84]. NREM sleep improves memory consolidation, reconsolidation, neurocognitive functions, or toxic waste removal. NREM stages, especially N3, control metabolic and hormonal activity—for instance, slow wave activity is responsible for maintaining glucose homeostasis (via improving insulin sensitivity), releasing growth hormone, or regulating lipid metabolism [85,86,87]. Overall, NREM sleep seems to be an essential stage, that through reduced energy expenditure, creates a favorable environment for maintaining homeostasis and restoring cell energy supplies.

### 2.2. REM

Another stage of human sleep is called REM (rapid eye movement) or paradoxical sleep. It is a phase originally discovered by Aserinsky and Kleitmann in 1953 [88]. REM is a part of sleeping time occurring every 90–120 min of sleep with some distinctive features, such as changes in electrical activity of the brain registered in EEG (increased cortical activation, occurrence of theta rhythm in hippocampus) or intermittent muscle twitches. Furthermore, the fluctuation in body temperature is another important feature of REM sleep. A natural initial drop in body temperature occurs during the transition into this stage, but once in REM sleep, the hypothalamus maintains core temperature stability, while peripheral cooling mechanisms, such as peripheral blood vessel dilation, contribute to the perception of cooler extremities, all of which are integral components of the REM sleep state. Another key characteristic of REM sleep is the presence of active muscle atonia and elevated arousal threshold [89].

The latter seems to be the most important in the theme of our review. During REM, the group of cells in the sublateral dorsal nucleus (SDL; ‘’Atonia Generator”) are being activated. They are GABAergic neurons located in the dorsal pontine brainstem. Due to their synaptic connections with interneurons of the ventral medulla and spinal cord, they are able to suppress the activity of alfa motoneurons. Interestingly enough, the same mechanisms are involved in the pathogenesis of cataplexy, narcolepsy, and other sleep–wake disturbances, and its pathologies are significant prognostic factors of some synucleinopathy [90].

Paradoxical sleep can be further subdivided into phasic and tonic phases. The phasic phase has some unique features: presence of eye movement linked to pontine-geniculate-occipital waves in EEG, contraction of middle ear muscle, or myogenic twitches of skeletal muscle. The heart rate and respiration become irregular. Moreover, a reduction in adrenergic activity is observed. The phasic stage of REM is probably the one with the most intensive dreaming experience [91]. 

Even though the tonic phase is linked with activation in EEG, during this period, we seem to be more alert, and arousal threshold is significantly lowered in comparison to the phasic phase [91]. Moreover, processing of external stimuli is significantly different during these phases with a reduction during phasic REM and a reappearance during tonic one. Dreams also can occur during tonic REM but they seem to be less vivid and abstract. Another important feature of the tonic phase of REM sleep is the appearance of alfa and beta frequency bands in EEG. Those findings suggest distinctive purposes of tonic phase, but this topic needs further investigation [92]. 

The biological function of REM is still not fully discovered, and new investigations bring rather new questions than new answers. For instance, it has been believed that muscle atonia during REM is a protection against acting out dreams. However, recent data show that dreaming can occur in every stage of human sleep, which throws this claim into question [93]. Attempts to solve the mystery of paradoxical sleep, lead to the so-called ontogenetic hypothesis. Considering that REM sleep time decreases in our life (8 h in infants vs. about 2 h in adults), some researchers believe that this part of sleep time is crucial to human brain genesis [94]. The duration of REM sleep correlates with the number of new synapses and constitutes a source of activation needed to provide convenient conditions for maturation of the central nervous system. Moreover, muscle twitches during this phase are needed for proper sensorimotor system development [95]. Those and other findings are pointing out the role of REM sleep in neurogenesis [96]. Recent studies also underline the neurofunctional importance of REM. The newest data point out its key role in these processes as emotional processing, spatial memory consolidation, neuroplastic, and even development of consciousness [97]. Another attempt to find REM sleep function is the so-called energy allocation hypothesis. This theory refers to the ability of all organisms to temporarily allocate the energy to maintain different basic functions in a manner that meets their temporary needs. Sleep–wake cycle plays an important role in reallocation of the energy needed to perform high-demand, wake-related activities compared to other essential biological functions. The role of REM sleep in this process includes suspension of thermoregulatory defense and decrease in skeletal muscle tone, which allows for the energy allocation necessary for sleep specific biological activities including cellular repair and neuronal network reorganization [98].

Fluctuation of motor activity during paradoxical sleep should also be considered when it comes to explaining its purpose. The level of motor activity during REM predicts its termination in rodents. It increases gradually and is highest just before transitioning to wakefulness [99]. It may suggest that this phase serves as an interlude, preparing the body for awakening. Moreover, people seem to be more alert after waking up from REM, which supports this hypothesis.

### 2.3. Polysomnography

**Figure 2:**
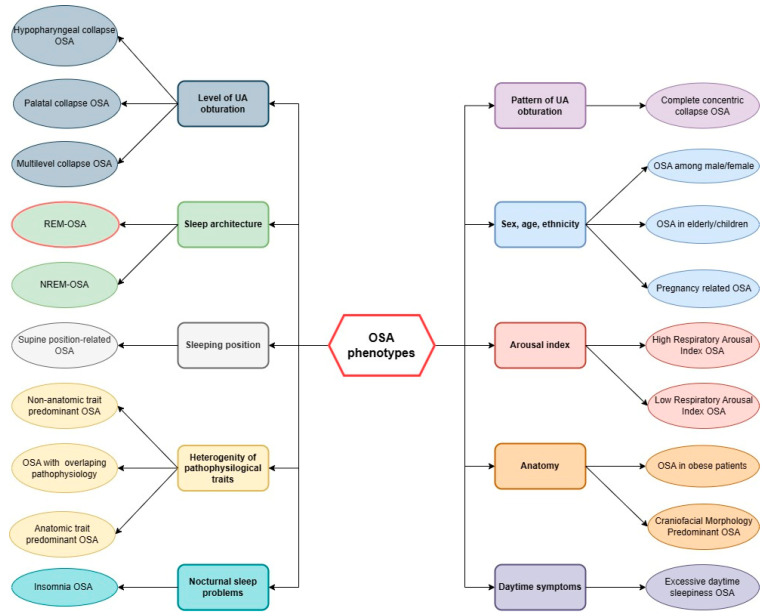
Examples of the most common phenotypes of obstructive sleep apnea (OSA). To subdivide OSA patients, the following characteristics can be used: sleep architecture (rapid eye movement-related OSA (REM-OSA) and non-rapid eye movement-related OSA (NREM-OSA)), level of upper airways (UA) obturation (palatal, hypopharyngeal, multilevel collapse of UA), pattern of UA obturation (e.g., OSA with complete concentric collapse of the palate), sex, age, and ethnicity (OSA in elderly/children, OSA in males/females, pregnancy-related OSA), arousal index (high-respiratory arousal index OSA, low-respiratory arousal index OSA), anatomy (OSA in obese patients, craniofacial morphology predominant OSA), presence of daytime symptoms (excessive daytime sleepiness OSA), presence of nocturnal sleep problems (insomnia OSA), and heterogeneity of OSA pathophysiological traits (categorization of OSA patients due to the predominant pathophysiology—anatomic or non-anatomic (e.g., arousal threshold, loop gain, muscle responsiveness)). Legend: NREM—non-rapid eye movement sleep, OSA—obstructive sleep apnea, REM—rapid eye movement sleep, UA—upper airways.

Polysomnography (PSG), as mentioned above, is a gold standard procedure for diagnosis of OSA. It also provides essential data to differentiate specific OSA phenotypes (see Figure 2). During the procedure, multiple physiological signals are recorded in the examined patient. PSG consists of several measurements, including electroencephalography (EEG), electrooculography (EOG), electromyography (EMG), electrocardiography (ECG), oxygen saturation, nasal/oral airflow, snoring, and chest and limb movement [100]. Patients with a presumptive diagnosis of OSA undergo the examination during sleep in the sleep lab where all the channels are set up. The data recorded during sleep are then collectively analyzed to determine the specific disorders. In terms of OSA and its phenotyping based on sleep stages, the most important measurements are EEG, EOG, nasal/oral airflow, and oxygen saturation. The general criterium for OSA diagnosis is based on apnea–hypopnea index (AHI), which represents an average number of apneas (reduction in airflow by ≥90% or cessation of breathing lasting ≥10 s) and hypopneas (reduction in airflow by ≥30% lasting ≥10 s along with either an oxygen desaturation >3% or an arousal lasting ≥10 s) per hour [100]. AHI is also used to assess the severity of OSA. The measurement of oxygen saturation measures the level of hypoxemia evoked by the respiratory events. Based on the EEG, the sleep brain activity is recorded. As previously described, sleep stages are represented by specific brain activity visualized on EEG, and therefore by the movement of eyes that is reflected on EOG. By combining the data from these polysomnographic channels, the relationship between sleep stage and the occurrence of apnea-hypopnea events can be assessed. The most commonly used approach to determine the OSA phenotypes related to sleep stages requires comparing the frequency of respiratory events represented as AHI during REM sleep (REM-AHI) and NREM sleep (NREM-AHI). The ratio of REM-AHI to NREM-AHI greater than 2 indicates that sleep disorder breathing is predominant during REM sleep. Additionally, some authors have proposed, that for a better assessment of the role of REM sleep on sleep breathing, we should include the criteria of at least 30 min of REM sleep. Moreover, to increase the precision of the distinction, it is suggested that we should use the following criterium of NREM-AHI fewer than five events/h and a REM-AHI of at least five events/h [101]. In terms of OSA phenotyping, it is important to assess the prevalence of respiratory events in specific sleep stages as precisely as possible.

## 3. REM-OSA Phenotype

Phenotyping is widely used in other respiratory diseases like COPD and asthma. The heterogeneity of these respiratory diseases prompted clinicians to divide patients into similar groups based on various features, e.g., symptoms of disease, quality of life, and outcomes. This approach was also used for OSA (see Figure 2). Classification of patients into phenotypes has some important goals, including optimization of the diagnostic process, better selection of patients for clinical trials, and personalization of treatment. Designing new diagnostic technologies led to the usage of genomic, molecular, and cellular factors in phenotyping. 

In this review, we focused on the REM-predominant OSA, which constitutes up to 26% of OSA patients [10]. There are at least two arbitrary ways to define this specific group of patients. Both are based on the apnea–hypopnea index (AHI) [102]. One of them recognizes REM-OSA when AHI during REM is at least twice as high as NREM-AHI. Defenders of this classification underline that patients assigned to REM-OSA according to this definition has some unique features (for instance, lower desaturation index compared to NREM-predominant OSA). However, this model is criticized for being very broad and blunting patient classification. The alternative one demands AHI during REM to be 5 or more, while NREM-AHI less than 5. These proposed classification criteria are not, however, mutually exclusive, having a common part. Additionally, the total REM duration must be at least 30 min throughout the whole sleep [62]. It was observed that the stricter definition of REM-predominant OSA, the lower arousal index, and similarly, the severity of the disease.

**Figure 3:**
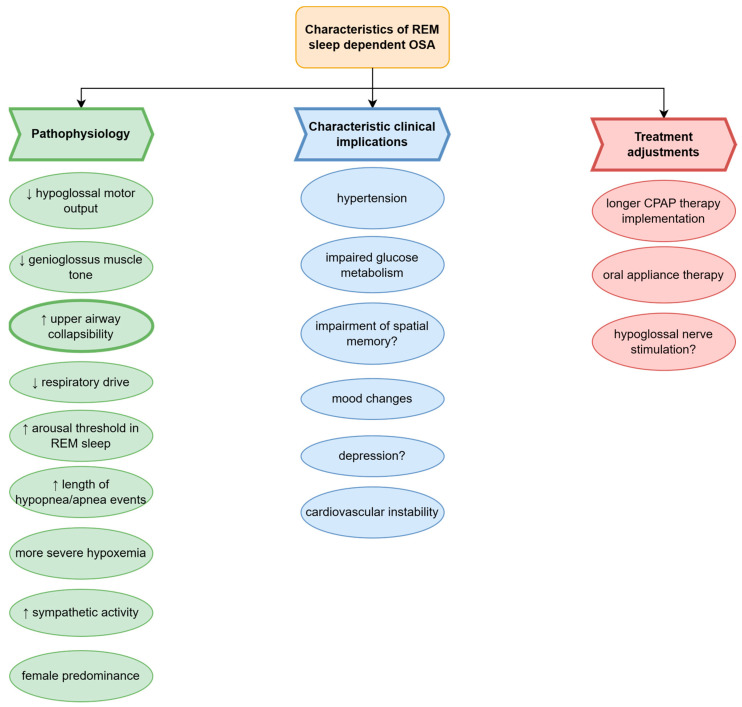
Characteristics of rapid eye movement (REM) sleep phase-related obstructive sleep apnea (OSA) phenotype. The pathophysiological and clinical features of OSA specific for REM-OSA phenotype. Pathogenesis of REM includes a decrease in hypoglossal motor output, and therefore decrease in genioglossus muscle tone during REM sleep leading to increased upper airway collapsibility. The described sequence of events in OSA patients is associated with more frequent and prolonged hypopnea-apnea events and severe desaturations during REM sleep. Additionally, REM-OSA has been described to increase sympathetic activity during REM sleep. The sleep phase-related OSA phenotypes may also be distinguished by clinical manifestations and comorbidities of OSA. REM-OSA has been described to contribute to cardiovascular instability, including increased risk of hypertension, impaired glucose metabolism, mood changes, depression, and possible impairment of spatial memory. Different endotypes underlying REM-OSA phenotype may influence the therapeutic approach. REM-OSA patients with increased upper airway collapsibility may benefit from long-er CPAP therapy to cover most of the REM sleep stages, oral appliance therapy, as well as hypoglossal nerve stimulation to increase the genioglossus muscle tone and prevent the upper airway collapse during REM sleep. Legend: OSA—obstructive, REM—rapid eye movement sleep.

Unique features and functions of REM sleep led to different courses of disease among REM-OSA patients (see Figure 3). Muscle atonia, involving upper airways increases the probability of its obstruction. In particular, diminished genioglossus activity, secondary to the cholinergic mediated inhibition of hypoglossal motor output, may be due to the fact that this muscle upholds the upper airways and supplies its patency; therefore, its decreased tension is often the chief culprit of REM-OSA [102].

Using positron emission tomography, researchers have found increased regional blood flow in both amygdalae during REM sleep [103]. Moreover, strong activation of the limbic system was observed. It is one of the reasons why this phase of sleep is believed to play a crucial role in emotional well-being. Observation of REM-OSA patients confirm this assumption—they more often struggle with depression and insomnia than other phenotypes [104]. Moreover, other symptoms of neurocognitive decline are noticed (e.g., in psychomotor speed) [105]. 

Patients with REM-OSA tend to have some common features. First, they are mainly younger females, often with symptoms of depression [102]. One of the possible explanations for this phenomenon is the reduction in the depression-protective effect of female sex hormones during REM. Both the quantity and efficiency of their sleep are certainly interrupted. The length of apnea-hypopnea events is significantly higher and related hypoxia is more severe. Apart from decreased activity of genioglossus muscle, lower respiratory drive is observed among this phenotype.

Moreover, most episodes of sleep disturbance in childhood happen during the REM phase, because it is the dominant phase in the early stage of development [106,107]. The same applies to obstructive sleep apnea [108]. This leads to the conclusion that REM-OSA is a reliable phenotype among children.

An escalating body of research is currently focused on elucidating the genetics and epigenetics of OSA. The genes under investigation within this context participate in a myriad of physiological mechanisms, for example, metabolic regulation, ventilatory modulation, craniofacial morphogenesis, or sleep cycle regulation [109]. Additionally, some allelic frequency is significantly associated with the AHI level, BMI, and obesity. On the other hand, intermittent hypoxia occurring in OSA leads to epigenetic alteration in genes responsible for systemic inflammation, which is part of the pathogenesis of many adverse effects like endothelial dysfunction or cardiovascular pathology [110]. Currently, there is a limited body of research investigating the genetics and epigenetics of the REM phenotype of OSA. In one study, variations in the FKBP prolyl isomerase 5 (FKB5) gene’s single nucleotide polymorphisms have been connected to respiratory incidents during REM sleep among individuals with moderate to severe OSA [111]. Moreover, the study conducted by Bartoluci et al. revealed a noteworthy observation, wherein the manifestation of OSA in mice was predominantly observed during the REM phase. Furthermore, this occurrence was notably pronounced in mice used as an established model of Down syndrome (Ts65Dn) [112]. It is also important to acknowledge that additional perturbations associated with REM sleep, such as REM sleep behavior disorder, have been correlated with diverse genetic anomalies [113]. These findings underscore the necessity for more extensive investigations in this domain to gain enhanced insights into the pathophysiology of REM-related OSA.

## 4. Non-Stage Specific OSA Phenotype

Sleep respiratory events in OSA may occur at different sleep stages. As previously described in sleep stage-related phenotypes of OSA, apneas/hypopneas predominantly occur during REM or NREM sleep. Although the REM-OSA phenotype is characterized by relatively high prevalence, the non-stage specific OSA (nssOSA) is more common in the general OSA population with a prevalence of around 66% of OSA patients [114]. Given that NREM sleep occupies the larger portion of total sleep time, it becomes apparent that events transpiring during this phase outnumber those arising during REM sleep. However, it is worth noting that the convergence of REM and NREM stages could potentially exacerbate the adverse effects of OSA. This is due to the distinct functional roles of these sleep stages, both of which are disrupted under these circumstances. This statement finds support in a study conducted by Chiu et al., wherein the non-stage specific OSA group exhibited a notable clustering of severe desaturation, primarily confined to REM sleep. Notably, this group displayed elevated OSA severity and a higher desaturation index in comparison to the REM specific OSA group [115]. Nonetheless, further investigations on this topic are warranted to deepen our comprehension. Although the nssOSA could also lead to several specific comorbidities, and is more common than sleep stage specific phenotypes, in our review, we aim to focus on the influence of disruption of specific sleep stages and OSA.

## 5. Clinical Significance of REM-OSA Phenotype

Similar clinical manifestations in OSA patients may be a result of different pathophysiological pathways responsible for sleep apnea. The recognition of various subtypes of OSA defined by distinct pathomechanisms has been a topic of extensive research in recent years [116]. Although it is important to acknowledge the various pathological mechanisms underlying OSA, the understanding of the differences between subtypes of OSA is crucial for the introduction of appropriate treatment and assessment of the comorbidity risk. The identification of REM-predominant OSA subtype is of great importance in terms of widely described clinical implications.

There has been a discussion that the REM-predominant subtype of OSA may be responsible for the more severe metabolic and cardiovascular complications. Characteristic pathomechanisms involved in the development of the OSA-REM subtype, such as increased sympathetic tone, increased upper airway collapsibility, and decreased respiratory drive, are described to play an important role in the development of several metabolic and cardiovascular disorders.

A large cohort study provides evidence that AHI in REM is, independently of non-REM-AHI, responsible for prevalent hypertension. Furthermore, its results showed that REM-AHI is significantly positively correlated with prevalent hypertension [117]. The Men Androgens Inflammation Lifestyle Environment and Stress (MAILES) provide data that severe REM-OSA (REM-AHI >30/h) is associated with prevalent and recent onset hypertension, while the association with non-REM-AHI has not been confirmed [118]. The possible pathomechanism underlying this correlation is still unclear. It has been hypothesized that increased sympathetic activity during REM sleep, in connection with sleep apnea-related increase in sympathetic tone, is responsible for the increase in blood pressure [66,119,120]. Longer and more severe apneas during REM sleep and associated lower nocturnal oxygen desaturation may also lead to hemodynamic changes [121,122]. The identification of REM-predominant subtype of OSA may be of great significance when it comes to OSA therapy in this group of patients. REM-OSA patients may benefit from longer CPAP therapy as REM sleep is more prevalent at later stages of sleep. In the randomized controlled trial, it has been shown that the number of hours spent in CPAP therapy is positively correlated with a decrease in 24 h of mean blood pressure [123]. There are clinical studies providing evidence that the number of hours of CPAP use at night is positively correlated with a decrease in blood pressure.

REM-predominant OSA may be linked with impaired glucose metabolism. In a prospective study, increased REM-AHI has been shown to be correlated with worse glycemic control in patients with T2DM measured as increasing levels of glycated hemoglobin (HbA_1c_) [124]. In contrast, NREM-AHI has not been significantly correlated with HbA_1c_ level. Sleep apnea during REM sleep may be also associated with nocturnal glucose levels. It has been described that the occurrence of sleep-disordered breathing during REM sleep results in the reversal of physiological decline in interstitial glucose concentration during REM sleep. In the NREM phase, sleep-disordered breathing has no significant effect on interstitial glucose concentration [125]. On the other hand, in another study, REM-AHI has been described to be significantly associated with insulin resistance assessed with HOMA-IR but not with fasting glycemia or glucose intolerance [126]. There are studies providing evidence about the molecular processes underlying impaired glucose metabolism in OSA during REM sleep. It has been suggested that the changes at the proteomic level in different OSA phenotypes may be responsible for differences in metabolic and cardiovascular outcomes. In a cohort study, authors described that severe OSA during REM sleep is associated with decreased plasma levels of Sirt2, LAP-TGF-β_1_, and Axin1—proteins involved in metabolic regulation and anti-inflammatory processes [127]. In terms of glucose metabolism, the protein of special interest is Sirt2, which is an NAD^+^-dependent deacetylase, that regulates many physiological functions. Moreover, it has been observed that decreased level of Sirt2 is present in insulin-resistant hepatocytes. On the other hand, the increased expression of Sirt2 happened to improve insulin sensitivity [128]. The authors of the same study concluded that increased Sirt2 expression in human peripheral blood mononuclear cells is negatively correlated with insulin resistance and diabetes [128]. Additionally, there are studies describing the correlation between increased Sirt2 expression and its suppressive function on adipocyte differentiation [129]. Taking these discoveries into consideration, we can assume that OSA during REM sleep may affect the exacerbation of metabolic disorders due to changes in proteomic level. Additionally, it has been described that CPAP therapy in REM-OSA patients, when elongated to cover the REM sleep phase, reduces HbA_1C_ [124]. Overall, existing studies support the thesis that REM-predominant OSA is associated with impaired glucose metabolism, although further research is needed to establish the possible treatment protocols to improve glycemic control in patients with coexisting REM-OSA and T2DM. 

One of the most common OSA comorbidity is neurocognitive function impairment manifesting as excessive daytime sleepiness and memory deterioration. There are a few clinical studies providing evidence that REM-AHI is not significantly correlated with the excessive sleepiness assessed with the Epworth sleepiness scale and multiple sleep latency test. In contrast, NREM-AHI shows a significant correlation with increased daytime sleepiness [130,131]. On the contrary, results of the latest study show that daytime sleepiness in a group of REM and NREM-OSA is similar, despite NREM-AHI being two times higher than REM-AHI, respectively, which may lead to the conclusion that daily sleepiness may be correlated with REM-AHI [132]. Although the impact of REM-OSA on daytime sleepiness is not very clear, the effect on memory impairment has been suggested. Animal model studies show that REM sleep insufficiency leads to spatial memory deterioration [133]. This thesis is confirmed in a human study with artificially induced REM-OSA. In this study, the authors withdrew CPAP therapy specifically in REM sleep, which resulted in severe apnea episodes during REM sleep. The improvements in maze testing, between nights with induced REM-OSA and nights of normal sleep, spent with a therapeutic time of CPAP, were both in completion time (mean of 31.3%), distance traveled (median of 34.3%), and distance spent backtracking (median of 56.5%) [134]. The results of this study show that disruption of REM sleep by sleep apnea leads to impairment of spatial memory, even though total sleep time has not been disturbed, and may be reversed by proper CPAP implementation [134]. Even though there are reports regarding the impact of REM-OSA on memory deterioration, the most important sleep phase for memory consolidation is NREM [135]. OSA during NREM sleep may affect memory processing to a greater extent than in REM sleep, although these differences may depend on the type of memory [105,136]. During physiological sleep, it is suggested in the literature, that REM sleep is responsible for emotional memory, while NREM sleep is attributed to declarative and procedural memory. The disruption of memory processes during OSA in specific sleep stages needs further investigation to evaluate memory outcomes.

Another important aspect of the OSA-REM subtype is its impact on mood changes. It has been observed in animal models that deprivation of REM sleep may evoke depression-like behavior in rats [137]. The current research on the link between REM-OSA and depression has given some interesting results. In the recent study, authors provided evidence that in the group of 166 OSA patients, subjects with REM-related OSA had significantly higher depression rates (assessed with Hospital Anxiety-Depression Scale) compared to NREM-OSA patients [138]. Additionally, another cross-sectional study of the Korean OSA population provides evidence that REM-OSA is positively associated with depressive symptoms in the men cohort; the authors have not observed a significant correlation in the female group [139]. The relationship between REM-OSA and depression may have many complex underlying pathomechanisms. The disruption of sleep architecture is one of the main symptoms of depression, and may be among the reasons for the greater prevalence of REM-OSA in the group of depressive patients. It has been described that depression is associated with disrupted REM sleep—shortened REM latency increased REM duration, and increased REM density [140]. The assumption could be made that, these changes in REM sleep may exist prior to the sleep-disordered breathing. Therefore, the episodes of sleep apnea could be more prevalent during REM sleep, in which duration is prolonged in depressive patients. Further research is needed to establish the pathophysiology of the correlation between REM-OSA and depression.

Taking into consideration all of the possible OSA complications that may be aggravated in a specific subtype of OSA such as REM-OSA, the recognition of the correlation between sleep architecture and apnea events may be beneficial to provide the best-personalized therapy for OSA and its comorbidities (see Figure 3). The proper diagnosis of OSA in terms of sleep phase is important to predict the most common complications correlated with the REM subtype of OSA. Moreover, the recognition of REM-OSA may influence the duration of CPAP treatment to cover the REM stage of sleep for better control of comorbid pathologies (see Figure 3). 

## 6. Treatment Adjustments in REM-OSA Phenotype

The gold standard treatment in OSA is continuous positive airways pressure therapy, as it offers the greatest efficiency and protective effect in terms of cardiovascular comorbidities [141,142,143]. Despite its effectiveness in reducing the outcomes of OSA, it is characterized by poor adherence in the general population of OSA patients [144]. Other existing therapeutic strategies for OSA include oral appliances (used to prevent the tongue and lower jaw from blocking the upper airway), hypoglossal nerve stimulation, and positional therapy. The possible treatment approaches for REM-OSA patients are discussed. One of the proposed hypotheses is implementing longer periods of CPAP to cover the later sleep stages mostly consisting of REM sleep. Adequate adherence to CPAP is considered to be 4 h of CPAP use per night [145]. It has been suggested that 4 h of sleep covers only about 40% of sleep-disordered breathing during REM; therefore, CPAP therapy exceeding 4 h to 7 h per night could be beneficial for OSA-REM patients, in terms of AHI reduction as well as better control of metabolic and cardiovascular comorbidities [124,146]. Although longer periods of CPAP seem to have better results in REM-OSA patients, it raises a question regarding the problems with long-term adherence to this adjustment. Given that it is crucial to include other techniques to achieve therapeutic goals for REM-OSA, it has been described that the use of oral appliance therapy such as mandibular advancement splints may reduce REM-AHI, especially in supine position, although in one study, the effect of oral appliance therapy has been shown in just 12% of REM-OSA patients [147,148]. Given the specific pathophysiology of REM-OSA and the decrease in genioglossus muscle tone during REM as a mechanism of upper airway collapse, it has been hypothesized that the hypoglossal nerve stimulator may be beneficial for REM-OSA patients. The results of a large cohort study of OSA patients treated with hypoglossal stimulator, show that treatment with hypoglossal nerve stimulation reduces REM-AHI [149]. This may lead to a conclusion, that for better effectiveness of OSA therapy in a group of REM-OSA patients, treatment should be personalized, using combined techniques for better adherence and control of both AHI and OSA comorbidities. To establish specific therapeutic schemes and guidelines for REM-OSA, further prospective research is needed.

## 7. Conclusions

Sleep architecture plays an important role in maintaining health homeostasis. The individual sleep phases are involved in the regulation of many physiological functions, such as respiratory control, glucose metabolism, cardiovascular stabilization, memory consolidation, and mood stabilization. Disruption of sleep architecture may impact the course of different sleep disorders including OSA. The relevance of distinguishing sleep stages of predominant OSA phenotypes, as well as OSA phenotyping in general, has been widely discussed in recent years. One of the most important OSA phenotypes is REM-related OSA—the phenotype correlated with many specific pathophysiological and clinical features. The unique physiology of REM sleep may evoke or aggravate respiratory disturbances during sleep; therefore, this leads to the development of OSA. It has been hypothesized that decreased hypoglossal motor output and decreased respiratory drive during REM sleep may evoke the sleep stage-dependent instability of upper airways. Apnea-hypopnea events specific to REM sleep are characterized by more severe hypoxemia compared to non-sleep stage specific OSA. The consequences of pathological changes in both sleep architecture and upper airway activity should be taken into consideration for a better understanding of the therapeutic targets in this OSA phenotype. To provide the most effective therapy for REM-OSA patients, we need to connect the knowledge about REM sleep with OSA pathogenesis. In the literature, a number of authors suggest that adjustment of the duration of CPAP treatment during the night so that it covers the latter stages of sleep may improve the outcomes of the therapy in this group of patients. Additionally, other techniques such as oral appliance therapy and preventing upper airway collapse, may be beneficial in REM-OSA. The positive therapy outcomes, and prevention of potentially serious comorbidities, by possibly low-cost treatment adjustments are the reason why distinguishing REM-related OSA patients is important in the general practice of sleep specialists. Taking everything into consideration, it is crucial to further investigate the complex pathological links between sleep architecture disruption and OSA, as well as to develop clinical guidelines for screening and treatment of REM-related OSA.

## Data Availability

No new data were created or analyzed in this study. Data sharing is not applicable to this article.
